# Interval discrimination across different duration ranges with a look at spatial compatibility and context effects

**DOI:** 10.3389/fpsyg.2014.00717

**Published:** 2014-07-08

**Authors:** Giovanna Mioni, Franca Stablum, Simon Grondin

**Affiliations:** ^1^École de Psychologie, Université LavalQuébec, QC, Canada; ^2^Department of General Psychology, University of PadovaPadova, Italy

**Keywords:** time discrimination, context effect, spatial compatibility, temporal intervals, manual responses, verbal responses

## Abstract

In the present study, a time discrimination task was used to investigate the effect of different contexts for intervals varying from 400 to 1600 ms. A potential time-space interaction was controlled, and participants used both manual responses (Experiments 1 and 2) and vocal responses (Experiment 3). Three ranges of durations were employed (short, middle and long), and within each range condition, three standard values were used (400, 700, and 1000 ms; 700, 1000, and 1300 ms; and 1000, 1300, and 1600 ms). Within each range, standard intervals were randomized (Experiments 1 and 3) or remained constant (Experiment 2) within a block of trials. Our results suggest that context influences time discrimination performances only when the temporal range under investigation is below 1300 ms and the temporal intervals varied within blocks. In the case of temporal intervals longer than 1300 ms, participants presented a tendency to respond “long” independently of the procedure used. Moreover, our results suggested that performances in a discrimination task are mainly influenced by the fact of varying standard durations within blocks, and not much by the time-space compatibility.

## Introduction

There are different signs in the time perception literature revealing the vulnerability of psychological time; indeed, different contexts move the output of temporal mechanisms in different directions. Amongst the contexts exerting influences on time estimation, there is the fact of dealing with different temporal intervals within a given investigation. An extensive literature shows that the duration of an event is not solely experienced on the basis of its temporal properties: attention (Zakay, 1998; Brown and Boltz, 2002; Grondin and Rammsayer, 2003; see also Zakay and Block, 1996), arousal and emotional levels (Angrilli et al., 1997; Mella et al., 2011; Droit-Volet et al., 2013; Grondin et al., 2014), and stimulus context (Barnes and Jones, 2000; McAuley and Jones, 2003; Jones and McAuley, 2005) can all affect the experience of time. Additionally, the time scale of the stimulus and the task used to measure participants' subjective duration have a great influence on the mechanisms involved in temporal processing (Gil and Droit-Volet, 2011; Mioni et al., 2014b).

When investigating time perception, a major concern is related to the temporal range under investigation. In the field of time perception, researchers have mainly used intervals in the range of 100 ms to a few seconds (Grondin, 2001, 2010). This temporal range is particularly important in humans because it involves processes from motor control, speech generation, playing music, and dancing to more complex processes like learning and decision making (Buhusi and Meck, 2005).

A general tendency in timing literature, mainly in neuroscience researches, is to emphasize a distinction between intervals above and below 1 s, which is based on differential pharmacological effects (Rammsayer, 2008) and on patient studies with various brain damages (see Ivry and Spencer, 2004; Meck, 2005; Mioni et al., 2014a; Piras et al., 2014). Moreover, researchers claimed that processing of smaller intervals is more sensory based, or benefits from some automatic processing, whereas the processing of longer intervals requires the support of cognitive resources (also see Lewis and Miall, 2003; Hellström and Rammsayer, 2004). Even if this “1-s” transition period remains somewhat arbitrary, there is certainly some turning point on the time continuum given the benefit one should expect from adopting an explicit counting strategy for processing long temporal intervals (Grondin et al., 2004; Grondin and Killeen, 2009a,b). Indeed, there are empirical reasons to believe that this transition occurs circa 1.2 s (Grondin et al., 1999), at least for the processing of auditory time intervals, the Weber fraction for time increasing for intervals longer than 1.3–1.5 s (Gibbon et al., 1997; Grondin, 2012, 2014).

However, processing temporal intervals cannot be independent from methodological issues. For example, in a typical time discrimination task, participants are required to judge the relative durations of two temporal intervals successively presented (first “standard” and second “comparison”). Presenting intervals successively induces some bias in the perceived duration of intervals, this effect being known as the time-order error (TOE). A positive TOE is observed when the first stimulus presented is over-estimated whereas a negative TOE is observed when the first stimulus is under-estimated, compared to the second. Researchers have explained the TOE as the result of a response bias or of a perceptual effect (Allan, 1977; Hellström, 1985, 2003; Eisler et al., 2008). Providing participants with information about correct responses (feedback) in time discrimination tasks eliminates the tendency for judging the second duration as longer than the first (Jamieson and Petrusic, 1975, 1978). Such a result is consistent with the view that TOE is a reliable perceptual effect and that practice with feedback leads participants to adopt biased decision criteria to overcome this effect.

Moreover, and as revealed by Vierordt's law (Vierordt, 1868; Lejeune and Wearden, 2009), when short and long intervals are presented within the same experimental context, shorter intervals tend to be overestimated and longer intervals are underestimated. The point in between, for which there is no constant error, is called the *indifference point*. The estimated value of the indifference point indeed depends on the durations used in the experiment (Eisler et al., 2008; Lejeune and Wearden, 2009).

Jones and McAuley (2005) tried to give a comprehensive explanation of temporal performance using time discrimination tasks. They reported interesting results describing local and global context effects. The authors pointed out that the temporal context systematically affects the perception of a temporal interval. The authors used an experimental setting in which a series of brief tones are presented, which determine an isochronous sequence of inter-onset intervals (Base IOIs). The Base IOI sequence precedes the two final pairs of stimuli (standard and comparison) that have to be compared. In this paradigm, the rate of the Base IOI sequence alters the perceived duration of the standard interval, producing a *local context effect* (Barnes and Jones, 2000; McAuley and Jones, 2003). The alteration of the perceived duration occurs because the local context sequence induces an internal periodicity that distorts participants' perception of the standard IOI in the direction of the Base IOI (over- or under-estimation according to the Base IOI). Barnes and Jones (2000) also reported that the rate of other sequences within the same session affects the perceived duration of the standard IOI, producing a global context effect (see also McAuley and Jones, 2003; Large, 2008).

Another, more recent line of investigation rather explains temporal performance from a time-space compatibility perspective (*mental time line*; Ishihara et al., 2008). These studies describe an association between temporal duration and the spatial position of the response keys on the keyboard: specifically, the congruity between spatial and temporal information along the “mental time line” may facilitate manual responses, which may yield a *spatial–temporal association of response codes* (STEARC) effect (Ishihara et al., 2008). Short temporal durations are associated with left space, and long temporal durations are associated with right space. The time-space interaction follows the idea that time, space, and numbers are processed by a common system (Walsh, 2003), a magnitude mechanism that codes information according to a quantitative representation, usually outlined as a left-to-right orientation continuum. Specifically for the temporal domain, in the context of time discrimination tasks, participants are presented with pairs of temporal intervals (standard duration presented first and comparison duration presented second) and have to judge if the second interval presented is longer (or shorter) than the standard. For time discrimination task, the presentation order is critical in the experimental setting. Higher accuracy is expected when the duration of the comparison stimulus is short and the “short” response ispositioned on the left side, compared to the condition in which the duration of the comparison stimulus is short and the “short” response is positioned on the right side. Opposite performance patterns are expected when the duration of the comparison is long; in fact, in this case, higher accuracy is expected when the right response key is associated with the “long” response, compared to the condition in which the “long” response is positioned on the left side (Conson et al., 2008; Ishihara et al., 2008; Bonato et al., 2012). Therefore, it is possible that the negative or positive TOE, often observed in time discrimination task, is partly caused by time-space compatibility rather than a memory or perceptual process related to the temporal interval under investigation.

In the present study, we first wanted to know if using different temporal contexts affects duration discrimination. In particular, we focused on “1-s” temporal interval and we included it within different temporal contexts or as the longest, the medium or the shortest standard temporal interval. In this way, it was possible to determine if participants' performance depends specifically on the temporal interval used or if it is modulated by the context within which this interval is included. Our use of context effect is inspired more by the work related to Vierordt's law than by the context effect suggested by Jones and McAuley's (2005). In fact, in Jones and McAuley's (2005) studies the influence of context on temporal performance was induced by the presentation of sequences of brief tones. In the present study, participants performed a time discrimination task in which the 1-s interval was included in blocks of trials, which include different temporal intervals (longer or shorter than 1-s temporal intervals).

A second aim of the present study was to investigate the time-space compatibility in order to understand the influence of response lateralization on temporal performances. For this purpose, participants were asked to respond manually (pressing a designed lateralized response key on the keyboard) or to respond orally. It was then possible to determine if the preference in responding short or long depends on the compatibility between temporal intervals (short-long) and position of the response keys (left-right), i.e., time-space compatibility.

## Experiment 1

### Methods

#### Participants

Fifty-six students from the University of Padova (Italy) were randomly assigned to one of three experimental groups: 20 participants in Group 1 (*M* = 21.70 years; *SD* = 0.97) for which standard durations lasted 400, 700, and 1000 ms; 18 participants in Group 2 (*M* = 21.56 years; *SD* = 1.50) for which standard durations lasted 700, 1000, and 1300 ms, and 18 participants in Group 3 (*M* = 22.00 years; *SD* = 1.84) for which standard durations lasted 1000, 1300, and 1600 ms. This and the following studies, were conducted in accordance with the Department of General Psychology guidelines, and all participants completed an informed consent form.

#### Materials

**Figure 1:**
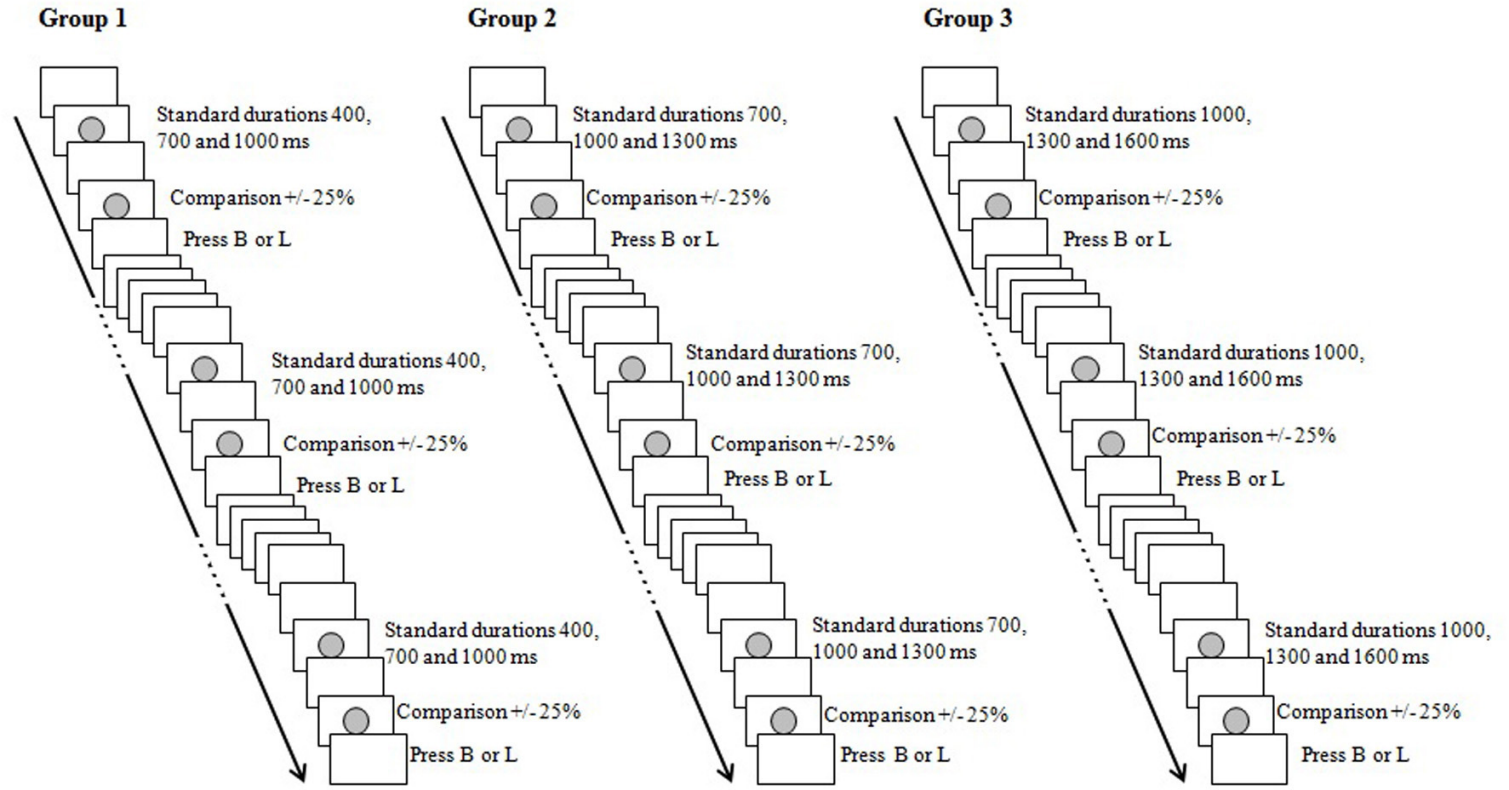
**Design of Experiment 1 (see Method)**. B, “Breve” (short in Italian); L, “Lungo” (long in Italian).

Each participant was tested in a quiet room at the Department of General Psychology of the University of Padova, (Italy). Each test session lasted approximately 20 min. All stimuli were presented on a 15-inch PC monitor and participants were seated at a distance of approximately 60 cm. The stimulus marking intervals to be discriminated was a gray dot centrally presented on a white background (Figure 1). We used E-Prime^®^ 2.0 to program and implement the tasks.

#### Procedure

Participants were instructed to complete a duration discrimination task by judging the relative duration of two time intervals successively presented. The first visual stimulus on the computer's screen marked the standard interval and the second one the comparison interval. For each group, three standard intervals were used. In Group 1 (short standard durations), the standards lasted 400, 700, and 1000 ms; in Group 2 (middle standard durations) they lasted 700, 1000, and 1300 ms; and in Group 3 (long standard durations) they lasted 1000, 1300, and 1600 ms. For each standard interval, one of two comparison stimuli was presented: ±25% with respect to the standard value (Table 1). Participants were seated at 60 cm from the computer screen and they were instructed to press two distinct keys: “B” if the second was shorter than the first one (“B” referred to the Italian word “Breve” = short) or “L” if the second was longer than the first one (“L” referred to the Italian word “Lungo” = long). For half of the participants the label with the letter “B” was placed over the letter “A” on the left of the keyboard and the label with the letter “L” was placed over the letter “L” on right of the keyboard; for the other half of the participants the label with the letter “B” was placed over the letter “L” on the left of keyboard and the label with the letter “L” was placed over the letter “A” on right of the keyboard. Twelve pairs of stimuli (standard—comparison) were presented within each block, and the standard durations were randomized within blocks. Stimuli sequences consisted of two gray circles separated by a 500-ms inter-stimulus interval; the next sequences of stimuli were presented 1000 ms after the participant's response. There were three blocks of trials in the experimental session. A practice phase was included at the beginning of the session in order to clarify the instructions and to familiarize participants with the task. One presentation of each pair of stimuli (standard—comparison) was included in the practice phase. Participants were instructed to be accurate and fast in their responses, and no feedback was provided.

**Table 1:** **Summary of standard and comparison temporal intervals used in Experiments 1–3**.

	**Group 1—short**		**Group 2—middle**		**Group 3—long**	
	**Standard**	**Comparison**	**Standard**	**Comparison**	**Standard**	**Comparison**
Standard-short	400	300	700	525	1000	750
		500		875		1250
Standard-middle	700	525	1000	750	1300	975
		875		1250		1625
Standard-long	1000	750	1300	975	1600	1200
		1250		1625		2000

#### Statistical analyses

Data were analyzed in terms of accuracy (proportions of correct responses) and perceived duration (proportion of “long” responses). For accuracy, an analysis of variance (ANOVA) according to a 3 (Group1—Short, Group 2—Middle, Group 3—Long) × 2 (Response key Short-left, Short-right) × 3 (Standard duration short, middle, and long) × 2 (Comparison short and long) design was conducted, with the Standard duration and Comparison being within-subject factors. An ANOVA on the proportions of “long” responses according to a 3 (Group1—Short, Group 2—Middle, Group 3—Long) × 2 (Response key Short-left, Short-right) × 3 (Standard duration short, middle, and long) design was conducted, the last factor being within-subjects.

To further investigate the effect of response key and spatial compatibility on time perception, we considered the responses as “congruent” when the comparison duration was short and the short response key was placed on the left side of the keyboard; and we considered the responses as “incongruent” when the comparison duration was short and the short response key was placed on the right side of the keyboard. Other ANOVAs were then conducted on proportions of correct responses according to a 3 (Group1—Short, Group 2—Middle, Group 3—Long) × 2 (Response key Congruent short-left, Incongruent short-right) × 2 (Standard duration short and long), the last factor being within-subjects. All significant analyses were followed by *post-hoc* analyses performed with a Bonferroni correction to reduce the Type I error rate, and the effect size was estimated with partial eta squared (η^2^_*p*_).

### Results and discussion

#### Proportions of correct responses

**Figure 2:**
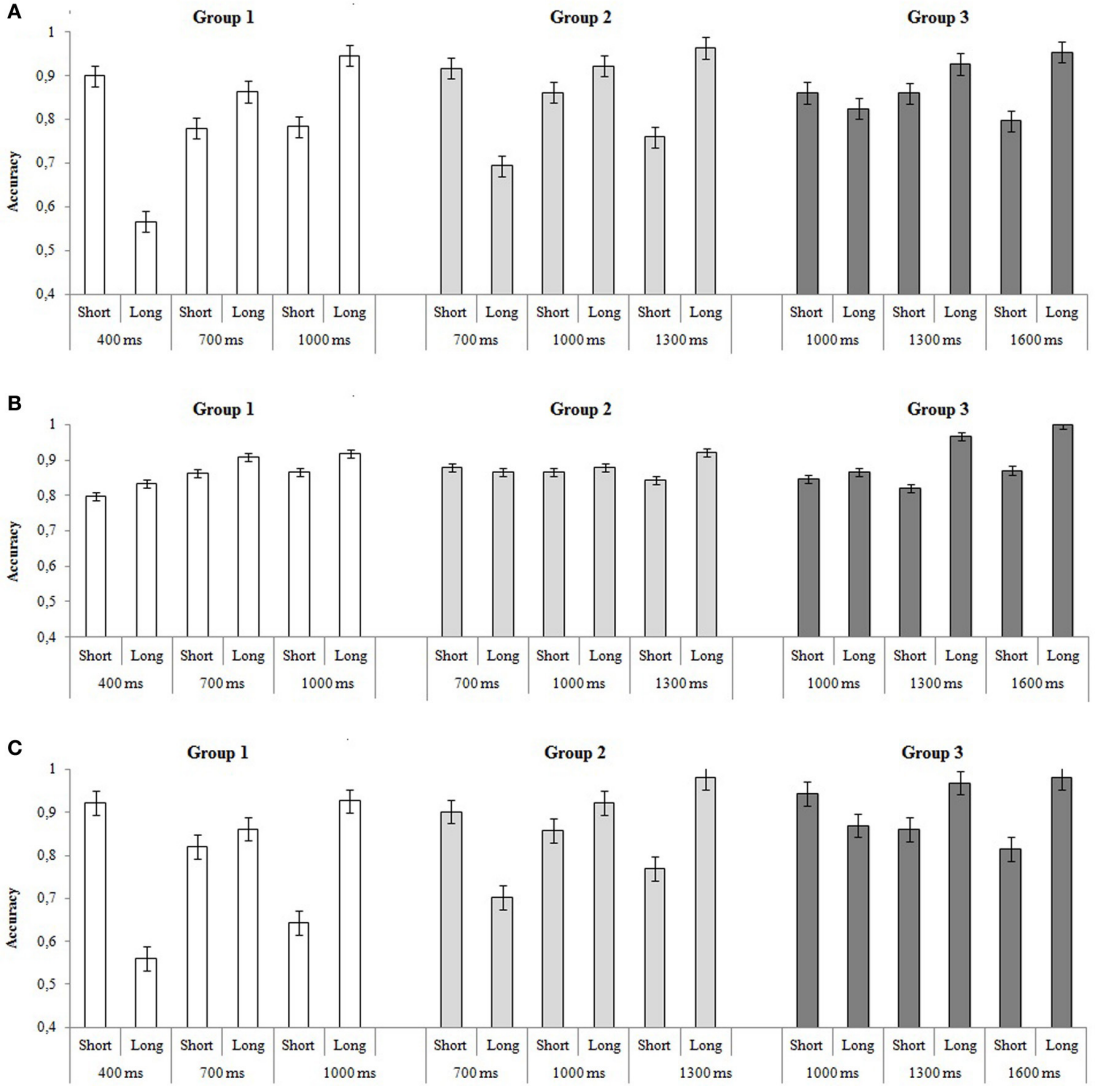
**Mean accuracy in each group as a function of standard durations and comparison intervals for (A) Experiment 1, (B) Experiment 2, and (C) Experiment 3**. The error bars indicate standard errors.

The mean proportions of correct responses as a function of groups, standard durations and comparisons are reported in Figure 2A. The ANOVA revealed a significant effect of group [*F*_(2, 53)_ = 5.44, *p* = 0.007, η^2^_*p*_ = 0.179] and standard duration [*F*_(2, 106)_ = 15.27, *p* < 0.001, η^2^_*p*_ = 0.234]. The analysis also showed a significant group × standard duration [*F*_(4, 106)_ = 2.49, *p* = 0.048, η^2^_*p*_ = 0.090] and standard duration × comparison interactions [*F*_(2, 106)_ = 64.49, *p* < 0.001, η^2^_*p*_ = 0.563].

Moreover, the group × standard duration × comparison interaction [*F*_(4, 106)_ = 4.93, *p* < 0.001, η^2^_*p*_ = 0.166] was also found (Figure 2A). *Post-hoc* analyses revealed similar patterns of performance in Group 1 and Group 2: participants were more accurate when the standard duration was short (standard-short = 400 ms in Group 1, and = 700 ms in Group 2) and when the comparison interval was shorter than the standard. No effect of comparison (standard-short vs. standard-long) was observed for the middle standard durations (standard-middle = 700 ms in Group 1 and = 1000 ms in Group 2) whereas, when the standard duration was long (standard-long = 1000 ms in Group 1 and = 1300 ms in Group 2), better performances were observed when the comparison was longer than the standard. In the case of Group 3, better performances were observed only when the standard was long (1600 ms) and when the comparison interval was longer than the standard.

No effect of comparison (*p* = 0.431) or response key (*p* = 0.104), as well as no interaction effect (all *p*s > 0.05), were significant.

#### Proportion of “long” responses

**Figure 3:**
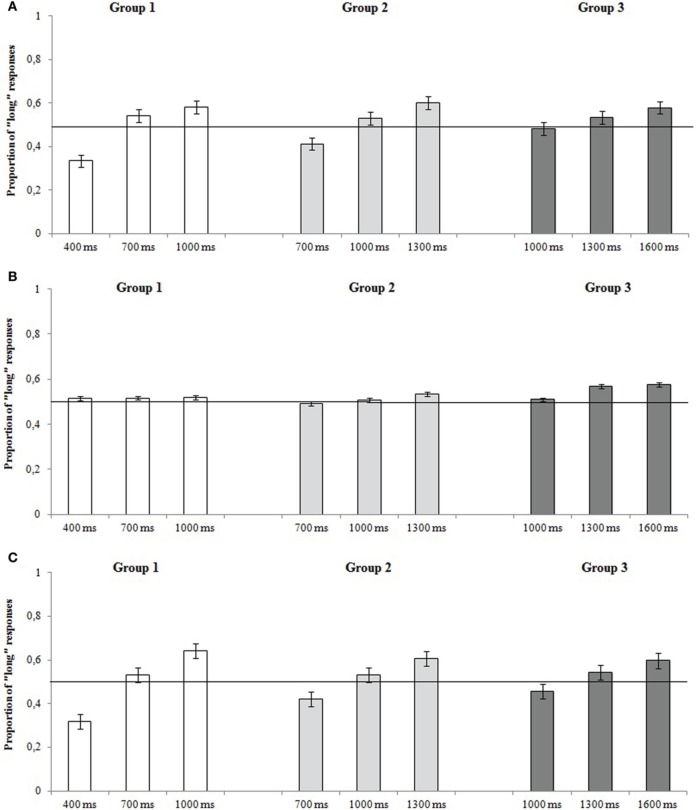
**Mean proportion of “long” responses in each group as a function of standard durations for (A) Experiment 1, (B) Experiment 2, and (C) Experiment 3**. The error bars indicate standard errors.

The mean proportion of “long” responses as a function of groups and standard durations are reported in Figure 3A. The ANOVA revealed a significant effect of standard duration [*F*_(2, 106)_ = 54.90, *p* < 0.001, η^2^_*p*_ = 0.523], and the group × standard duration interaction [*F*_(4, 106)_ = 4.53, *p* = 0.002, η^2^_*p*_ = 0.153]. *Post-hoc* analyses revealed that participants in all groups had a tendency in responding “long” when the standard duration was the longer presented. Participants of Group 1 (short = 400 ms) responded “long” less often when the standard duration was short than participants of Group 3 (short = 1000 ms). No effect of group (*p* = 0.180) or of response key (*p* = 0.870), or other interactions, were significant (all *p*s > 0.05).

#### Time-space compatibility (congruent vs. incongruent)

**Figure 4:**
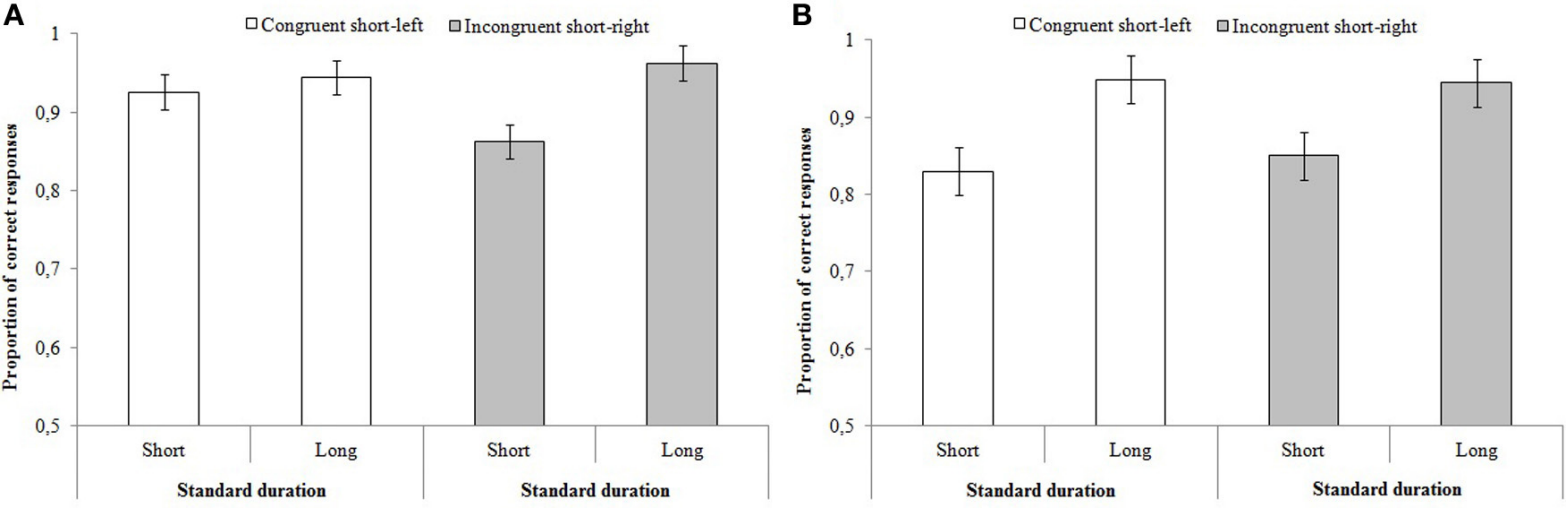
**Mean proportion of correct responses as a function of standard durations and response keys for (A) Experiment 1 and (B) Experiment 2**. The error bars indicate standard errors.

The analyses conducted to investigate the effect on accuracy of time-space compatibility (congruent vs. incongruent) revealed a significant effect of standard duration [*F*_(1, 56)_ = 11.59, *p* < 0.001, η^2^_*p*_ = 0.188] and a significant interaction between standard duration × response key [*F*_(1, 56)_ = 5.36, *p* = 0.025, η^2^_*p*_ = 0.097] (Figure 4A). *Post-hoc* analyses showed that participants were less accurate when the standard duration was short and the response key was on the right side but no effect of response key was found for long responses. No effect of group (*p* = 0.891) or of response key (*p* = 0.227), or other interactions, were significant (all *p*s > 0.05).

The results showed different performance patterns, depending on the temporal intervals under investigation. In the case of Group 1 and Group 2, results showed a positive TOE when the standard intervals were the shortest (400 ms in Group 1 and 700 ms in Group 2) and the comparison was shorter than the standard; whereas a negative TOE was observed when the standard interval was the longest of the experimental setting (1000 ms for Group 1 and 1300 for Group 2) and the comparison was longer than the standard. In the case of Group 3, a general tendency in responding “long” produced a negative TOE independently of the standard duration (Figure 2A).

These results are consistent with the idea that there is some transition regarding the temporal processes operating with different duration ranges in the vicinity of 1200–1300 ms (Grondin et al., 1999). Further analyses of the influence of the position of the response key on temporal performance revealed lower performances when the standard duration was short and the response key was in the incongruent condition (short-right).

## Experiment 2

In order to further distinguish the effect of context from the effect of temporal interval, the standard duration was kept fixed within blocks in the present experiment.

### Methods

#### Participants

As in Experiment 1, 56 students (*M* = 22.18 years; *SD* = 1.72) from the University of Padova (Italy) were randomly assigned to one of three experimental groups: 20 participants in Group 1 for which standard durations lasted 400, 700, and 1000 ms; 18 participants in Group 2 for which standard durations lasted 700, 1000, and 1300 ms, and 18 participants in Group 3 for which standard durations lasted 1000, 1300, and 1600 ms. All participants provided informed consent to complete the study and none took part in Experiment 1.

#### Procedure and materials

**Figure 5:**
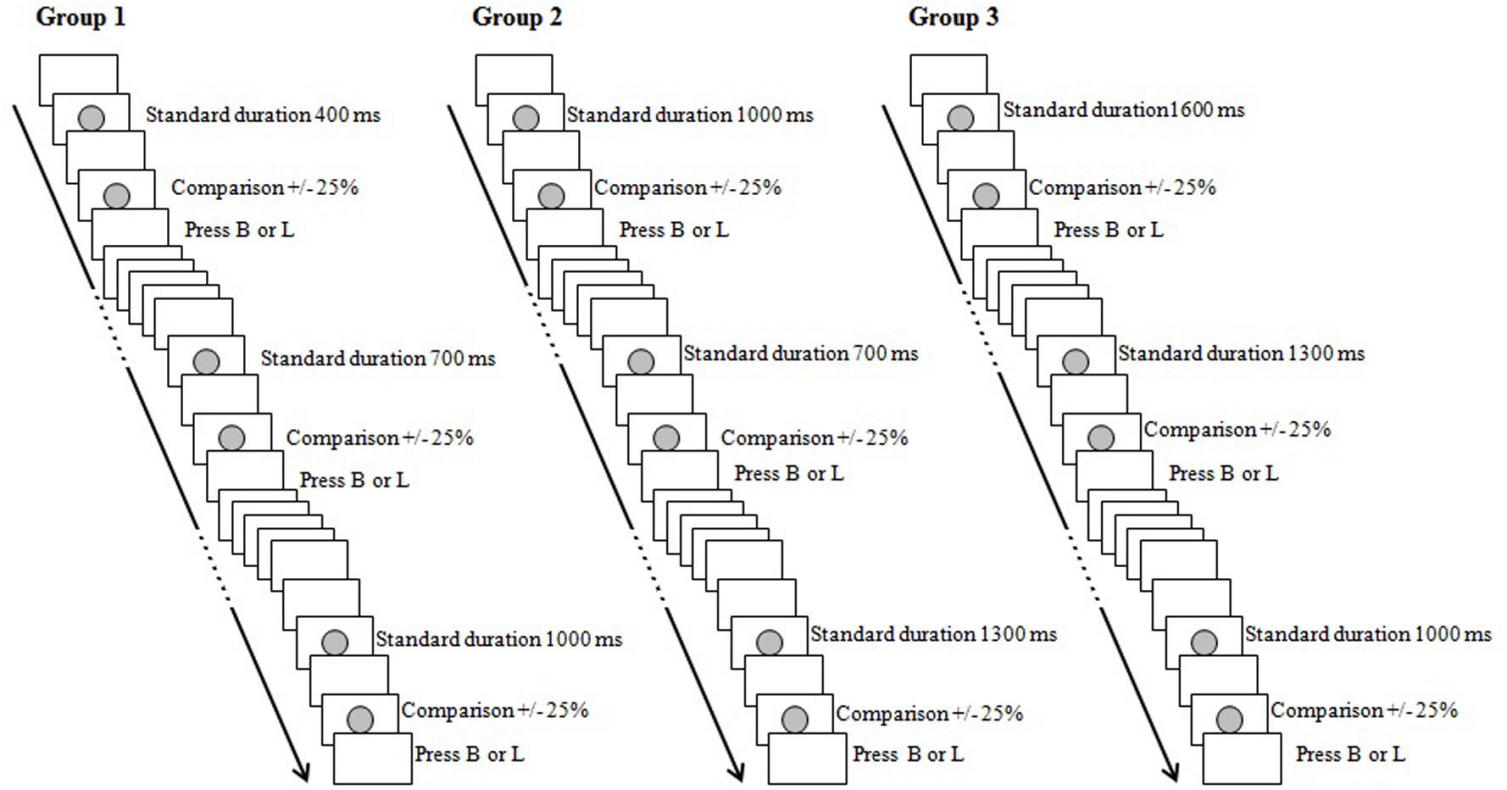
**Design of the Experiment 2 (see Method)**. B, “Breve” (short in Italian); L, “Lungo” (long in Italian).

The experimental setting was similar to the one used in Experiment 1 with one key difference: the standard durations did not vary within blocks (Figure 5). In each block, participants were always presented with the same standard duration and the comparison interval was ±25% compared to the standard (Table 1). The presentation order of the blocks was randomized. As in Experiment 1, there was a practice phase and no feedback; participants were instructed to be accurate and fast in their responses. The response keys were counterbalanced between participants. Finally, the designs of the statistical analyses are the same as the ones described in Experiment 1.

### Results and discussion

#### Proportions of correct responses

The mean proportions of correct responses as a function of groups, standard durations and comparison intervals are reported in Figure 2B. The ANOVA revealed a significant effect of standard duration [*F*_(2, 106)_ = 6.27, *p* = 0.003, η^2^_*p*_ = 0.111] and of comparison interval [*F*_(1, 53)_ = 12.42, *p* < 0.001, η^2^_*p*_ = 0.199]. No main effect of group (*p* = 0.360), response key (*p* = 0.710) or other interactions were significant (all *ps* > 0.05).

#### Proportions of “long” responses

The mean proportions of “long” responses as a function of groups and standard durations are reported in Figure 3B. The ANOVA revealed only a significant standard duration effect [*F*_(2, 106)_ = 3.03, *p* = 0.050, η^2^_*p*_ = 0.054] indicating that participants pressed “long” more frequently when the standard duration was the longest presented, compared to the shortest (*p* > 0.05 for all other effects).

#### Time-space compatibility (congruent vs. incongruent)

The analyses conducted on accuracy to investigate the effect of time-space compatibility (congruent vs. incongruent) (Figure 4B) revealed only a significant standard duration effect [*F*_(1, 53)_ = 19.02, *p* < 0.001, η^2^_*p*_ = 0.276] indicating that participants were more accurate when discriminating long temporal intervals. No other main or interaction effects were significant (all *ps* > 0.05).

Results from Experiment 2 showed high accuracy in all three Groups and a tendency in responding long when the standard duration was long and the comparison was longer than the standard. No effect of response key was observed, suggesting that this effect depends on the context in which the temporal interval is presented and is not specifically related to the single duration employed.

## Experiment 3

Pieces of scientific literature consistently suggest that there is an interaction between time and space (Ishihara et al., 2008; Bonato et al., 2012). In order to disentangle the specific effect of time-space compatibility from the effect of the temporal intervals employed, participants in Experiment 3 were asked to give vocal responses.

### Methods

#### Participants

As in Experiments 1 and 2, 51 students (*M* = 23.16 years; *SD* = 2.57) from the University of Padova (Italy) were randomly assigned to one of three experimental groups: 18 participants in Group 1 for which standard durations lasted 400, 700, and 1000 ms; 17 participants in Group 2 for which standard durations lasted 700, 1000, and 1300 ms, and 16 participants in Group 3 for which standard durations lasted 1000, 1300, and 1600 ms. All participants provided informed consent to complete the study and none took part in Experiments 1 or 2.

#### Procedure and materials

The material and procedure were exactly as in Experiment 1 (see Figure 1), except that participants were instructed to respond vocally by saying “Breve” (“Breve” = “short” in Italian) or “Lungo” (“Lungo” = “long” in Italian), instead of pressing the response keys.

### Results and discussion

Given that participants were instructed to give vocal response, no effect of response key is taken into consideration. Data were analyzed in terms of accuracy (proportions of correct responses), and perceived duration (proportion of “long” responses). For accuracy, an ANOVA according to a 3 (Group1—Short, Group 2—Middle, Group 3—Long) × 3 (Standard duration short, middle, and long) × 2 (Comparison short and long) design was conducted, with the Standard duration and Comparison being within-subjects factors. An ANOVA on the proportions of “long” responses according to a 3 (Group1—Short, Group 2—Middle, Group 3—Long) × 3 (Standard duration short, middle, and long) design was conducted, the last factor being within-subjects.

#### Proportions of correct responses

The mean proportions of correct responses as a function of groups, standard durations and comparison intervals are reported in Figure 2C. The ANOVA revealed a significant effect of group [*F*_(2, 48)_ = 19.85, *p* < 0.001, η^2^_*p*_ = 0.453] and of standard duration [*F*_(2, 96)_ = 7.72, *p* < 0.001, η^2^_*p*_ = 0.139]. The analyses also revealed a significant standard duration × comparison interval interaction [*F*_(2, 96)_ = 63.21, *p* < 0.001, η^2^_*p*_ = 0.568] and a significant group × standard duration × comparison interval interaction [*F*_(4, 96)_ = 4.59, *p* = 0.002, η^2^_*p*_ = 0.161] (Figure 2C).

Post-hoc analyses revealed that in Group 1 and Group 2, participants were more accurate when the standard duration was short (standard = 400 ms in Group 1 and standard = 700 ms in Group 2) and the comparison interval is shorter than the standard. An opposite pattern of performance is observed when the standard durations are long (standard = 1000 ms in Group 1 and standard = 1300 ms in Group 2). In this case, better performances are observed when the comparison is longer than the standard. No effect of comparison interval was observed for the middle standard duration (standard = 700 ms in Group 1 and = 1000 ms in Group 2). In the case of Group 3, better performances are observed only with the middle and long standard durations (1300 and 1600 ms) when the comparison interval is longer. No main effect of comparison (*p* = 0.176) or other interactions were found (all *ps* > 0.05).

#### Proportions of “long” responses

The mean proportions of “long” responses as a function of groups and standard durations are reported in Figure 3C. The ANOVA revealed a significant effect of standard duration [*F*_(2, 96)_ = 62.77, *p* < 0.001, η^2^_*p*_ = 0.567], as well as the group × standard duration interaction [*F*_(4, 96)_ = 4.14, *p* = 0.004, η^2^_*p*_ = 0.147] indicating that participants in Group 3 had a greater tendency to respond “long” for short standard intervals (Group 3 = 1000 ms) than participants in Group 1 (Group 1 = 400 ms). No main effect of group was found (*p* = 0.340).

The results observed in Experiment 3 are consistent with the ones reported in Experiment 1. In both experiments, the standard durations were randomly presented within blocks. Whether vocal or manual responses are used, participants showed a positive TOE when the standard intervals was shorter (400 ms in Group 1 and 700 ms in Group 2) and the comparison was shorter than the standard; a negative TOE was observed when the standard interval was the longest of the experimental setting (1000 ms for Group 1 and 1300 for Group 2) and the comparison was longer than the standard. In the case of Group 3 a general tendency in responding “long” produced a negative TOE independently of the standard duration was observed.

Participants' performance was not modulated by the assignment of response keys, thus suggesting that processing duration in time perception did not involve spatial representation. This finding would not follow predictions of time-space compatibility, but would rather be consistent with alternative interpretation frameworks. In particular, the present data would be consistent with a sequential order system that represents items with respect to other items, via an inter-item association or on the basis of their ordinal position in the sequence without an absolute, spatially-defined reference (Marshuetz, 2005).

## General discussion

The present study was conducted for testing the effect of various temporal contexts and the effect of time-space compatibility on duration discrimination. In particular, we focused on 1-s temporal interval considering that below and above this duration, different processes (automatic vs. controlled) would be at play (Lewis and Miall, 2003; Hellström and Rammsayer, 2004; Rammsayer, 2008).

### Temporal contexts

Results from Experiment 1 showed that participants' accuracy (percentage of correct responses) on time discrimination task depended on the stimulus duration and context. Interestingly, participants in Group 1 and Group 2 had a similar pattern of performance. In both cases, participants of these groups had a preference in responding “short” when the standard interval was the shortest, and in responding “long” when the standard duration was the longest and, this was true independently of the standard duration used. In fact, let's consider the standard intervals used for Group 1 and Group 2 (Table 1). Both groups had 700 and 1000 ms as standards, but in the case of Group 1, 700 ms was the middle and 1000 ms was the longest standard interval, and for Group 2, 700 ms was the shortest and 1000 ms was the middle standard interval. In the case of Group 1, no effect of standard-comparison was observed for the 700 ms standard interval (middle standard) and when the standard was 1000 ms (longest standard), participants had the tendency of responding “long.” But in the case of Group 2, participants had a tendency in responding “short” when 700 ms was presented (shortest standard) and no effect of standard-comparison was observed for 1000 ms (middle standard). A different pattern of performance was observed in Group 3, in which participants had the tendency of responding long, and this pattern occurred for the middle and the longest standard durations (Group 3 middle = 1300 ms and longest = 1600 ms).

Although it is difficult to totally discard the possibility that the temporal performances observed in Groups 1 and 2 are influenced by a perceptual phenomenon like the TOE (Hellström, 1977, 1978), a cognitive interpretation is viable. Over several trials, participants might have created anchor duration, i.e., a memory representation issued from the averaging of the shorter and the longer temporal intervals presented. It is posited that this anchor duration may exert influence (some weight) on the discrimination process at the moment of task (for a given single trial). Stimuli are partly classified as a function of the “anchor,” with stimuli below the “anchor” tending to be assigned as short and stimuli above the “anchor” as long (see also Oshio et al., 2006).

However, this anchor hypothesis does not explain the performance observed in Group 3. In fact, participants in Group 3 generally responded long independently of the duration of the standard interval presented. Such a result is compatible with the hypothesis stipulating that there are distinct systems for processing duration above or below 1-s, one for longer temporal intervals (in the range of seconds) and one for short temporal intervals (in the range of milliseconds). It is as if the amount of information to be processed exceeds the capacity of the system, just like there is a limited capacity of processing in working memory (Miller, 1956; Cowan, 2001), a limitation that could be compensated by re-organization of information processing with the creation of chunks of information. Actually, a spontaneous way for re-organizing temporal information (too long intervals) is to use strategies such as explicit counting or tapping (Grondin et al., 1999, 2004). In sum, the results of Experiment 1 suggest that, for duration between 400 and 1300 ms, temporal performance is influenced by the context, but for standard durations longer than 1300 ms, other processes seem to be involved and they would attenuate or erase the anchor effect reported with briefer intervals.

Experiment 2 showed that temporal performance is not only related to the range of the temporal intervals under investigation, but also to the experimental procedure used. In fact, without a randomization of trials, there is no anchor effect for short temporal intervals (Group 1 and Group 2). Interestingly, participants in Group 3 rather showed the same pattern of performance (preference in responding long) in Experiments 1 and 2. Moreover, for Group 3 in these two experiments, the accuracy level was about the same. This fact provides additional support to the idea that a different temporal information system would contribute to the processing of longer temporal intervals.

### Time-space compatibility

In the present study, we also tested whether time-space compatibility may influence a duration discrimination performance. A recent line of research explains temporal performance from a time-space compatibility perspective (Ishihara et al., 2008; Bonato et al., 2012). These studies suggest that humans do not process time and space separately, but represent time as space. Time flows using a spatial organization or a “mental time line.” In the present study, time-space compatibility would be expressed by an association between temporal duration and the spatial position of the response keys on the keyboard: specifically, short temporal durations would be associated with left space, and long temporal durations with right space. Such an association should lead to shorter reaction times and higher accuracies in the congruent (i.e., short-left) than in the incongruent (i.e., short-right) condition (Vallesi et al., 2008).

Results from our study tend to show at first sight that participants' performance could be modulated by time-space compatibility. In fact, in the case of Experiment 1, participants were less accurate when response keys were placed in the incongruent position (long-left and short-right), but only when the standard duration was the shortest. No effect of position of response keys on time discrimination performance was observed when the standard was the longest.

However, in the case of Experiment 2, there was no sign of a time-space compatibility effect on time discrimination performances. Participants were generally more accurate when discriminating long standard intervals, with no effect of position of the response keys. Results from Experiments 1 and 2 indicate the importance of comparing duration intervals with each other. In brief, the potential influence of space compatibility in time discrimination was only observed in Experiment 1, i.e., when the standard duration varied within blocks. Therefore, it is the implicit comparison of temporal intervals and not the duration of the interval itself that seems to be the key factor underlying the response bias (time-space compatibility) (Vicario, 2011).

Interestingly, in Experiment 3, in which no manual response was involved, participants showed exactly the same pattern of performances as the one observed in Experiment 1. In fact, participants had a tendency to respond “short” when the shortest standard duration was presented and “long” when the longest standard duration was presented. Participants' performance was not modulated by assignment of response keys, thus suggesting that processing of duration in time discrimination did not involve necessarily spatial representation. In other words, considering that the effect obtained in Experiment 1 (1) disappeared in Experiment 2 without the randomization of standard durations within blocks, but (2) occurred in Experiment 3 with this randomization but without the assignment of responses keys, what could have look like a time-space compatibility effect in Experiment 1 is indeed due to another factor, namely, varying standard durations within blocks.

It is difficult to compare the present findings with previous results, or to evaluate directly the impact of our findings on the material available in the literature. Previous studies used auditory stimuli and different experimental methods [see (Bonato et al., 2012) for a review]. It remains possible that a time-space effect is involved in other temporal performances, but this might be caused by specific methodological and/or strategic conditions rather than by a general and stable cognitive effect.

## Conclusion

In conclusion, we have found a complex interaction between context, time-space compatibility and temporal range under investigation. Our results suggest that context influences time discrimination performances only when the temporal range under investigation is below 1300 ms and the temporal intervals vary within blocks. In the case of temporal intervals longer than 1300 ms, participants presented a tendency to respond “long” independently of the method used to present the standard temporal intervals (intervals varying within blocks or between blocks). Overall, these findings indicate that distinct temporal processes might be at play above and below 1300 ms.

### Conflict of interest statement

The authors declare that the research was conducted in the absence of any commercial or financial relationships that could be construed as a potential conflict of interest.
